# ChIPOTle: a user-friendly tool for the analysis of ChIP-chip data

**DOI:** 10.1186/gb-2005-6-11-r97

**Published:** 2005-10-19

**Authors:** Michael J Buck, Andrew B Nobel, Jason D Lieb

**Affiliations:** 1 Department of Biology and Carolina Center for Genome Sciences, CB 3280, 202 Fordham Hall, University of North Carolina at Chapel Hill, Chapel Hill, NC 27599-3280, USA; 2 Department of Statistics, University of North Carolina at Chapel Hill, Chapel Hill, NC 27599-3260, USA

## Abstract

ChIPOTle (Chromatin ImmunoPrecipitation On Tiled arrays) takes advantage of two unique properties of ChIP-chip data: the single-tailed nature of the data, caused by specific enrichment but not specific depletion of genomic fragments; and the predictable enrichment of DNA fragments adjacent to sites of direct protein-DNA interaction. Implemented as a Microsoft Excel macro written in Visual Basic, ChIPOTle uses a sliding window approach that yields improvements in the identification of *bona fide *sites of protein-DNA interaction.

## Abstract 2

ChIPOTle is a new software tool designed specifically for the analysis of ChIP-chip data.

## Rationale

Interactions between proteins and DNA facilitate and regulate many basic cellular functions, including transcription, DNA replication, recombination, and DNA repair. For example, the process of transcription is regulated by a class of proteins referred to as transcription factors, which often bind to specific DNA sequences upstream of gene coding regions. This control mechanism allows cells to respond to developmental or environmental signals by using the same transcription factor to coordinate expression of many genes. Therefore, it is of interest to determine where regulatory proteins of this and other types are bound to the genome.

**Figure 1:**
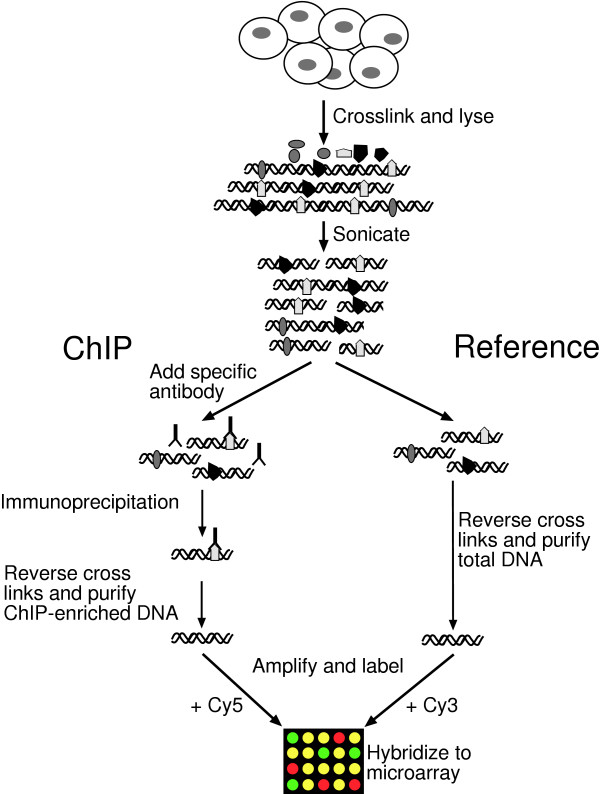
A summary of the ChIP-chip procedure. See the text for details.

The genomic-binding location of transcription factors can be determined using chromatin immunoprecipitation (ChIP) followed by detection of the enriched fragments by DNA microarray hybridization. This procedure, also known as ChIP-chip, has been reviewed extensively [1-5]. To appreciate the unique properties of the data generated by the ChIP-chip procedure, it is useful to review briefly the main points of the experimental procedure (Figure 1).

After growing the cells of interest under the desired conditions, chromatin is usually cross-linked with formaldehyde to preserve sites of interaction between proteins and DNA. The cross-linked chromatin is then sheared by sonication or enzymatic digestion. Shearing creates a population of chromatin fragments of varying size, generally ranging from 200 to 1,000 base-pairs. The protein of interest, along with the DNA associated with it, is then isolated by using an antibody specific to that protein or by affinity purification utilizing an epitope or affinity tag fused to the protein. The ChIPed DNA is then purified. Because yields from most samples are low, amplification is often required. DNA fragments enriched in the procedure are then detected by comparative hybridization to a DNA microarray. Standard technical recommendations common to all microarray experiments (for example, the need for dye swaps) apply equally to ChIP-chip experiments. The result of the hybridization allows one to identify which segments of the genome were bound by the protein of interest during immunoprecipitation.

**Figure 2:**
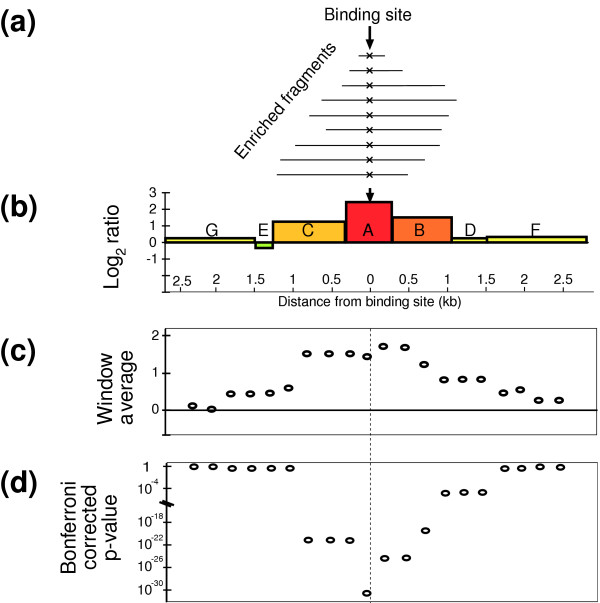
The neighbor effect and calculation of *P *values. **(a) **After ChIP, purified DNA fragments bound by the protein of interest will be of various lengths. **(b) **Actual log_2 _ratios reported by arrayed elements for Rap1p binding to promoter region of RPL1B (array element 'A') from the Rap1p binding dataset reported by Lieb and coworkers [13]. Arrayed element 'A' contains the actual site of protein-DNA interaction, and so this spot will have the highest ratio (red = high positive ratio; yellow = low ratio; green = negative ratio). Arrayed elements 'B' (RPL1B open reading frame [ORF]) and 'C' (MRM2 ORF), which are within about 1 kilobase (kb) of the binding site, are also enriched above noise. Arrayed element 'B' has a higher ratio then spot 'C', because the binding site is located closer to element 'B'. The arrayed elements 'D', 'E', 'F', and 'G' are too far from the binding site to be enriched. **(c) **Using a 1 kb window with a 0.25 kb step, the value of each window is plotted. The location of each window is defined by its central coordinate. **(d) **The *P *value of each window is plotted. The Bonferroni corrected *P *values were calculated based on the observed data, which had a log_2 _background standard deviation of 0.32 with 21,208 comparisons. Note that the window with the smallest *P *value (about 10^-30^) does not correspond to the highest window average. This is due to the fact that the most significant window contains three arrayed elements (A, B, and C), whereas the windows with the highest average contain only two elements (A and B). In this case, the center of the window with the highest *P *value is located about 80 bases from the actual binding site.

The interpretation of data generated by a ChIP-chip experiment is in many respects similar to interpretation of traditional gene expression microarrays, but it differs in two important ways. First, in traditional expression experiments, each element on the microarray measures the abundance of RNA molecules of a fixed length. (Note that we shall use the term 'arrayed elements' hereafter to describe DNA fragments that are deposited on the surface of the array; the term 'probe' is sometimes used by others.) In contrast, with ChIP-chip experiments each element measures the abundance of a population of fragments of various lengths due to the effects of chromatin shearing. As a consequence, arrayed elements representing genomic regions both at the binding site and near the binding site will detect enrichment (Figure 2).

Depending on the method and degree of chromatin shearing, and the resolution of the arrayed elements, this effect produces a 'peak' of signal centered over the binding site, which may span several arrayed elements representing genomically adjacent DNA. This 'neighbor effect' is not an expected property of noise or other spuriously high ratio measurements, and thus is a source of information that can be used for analysis.

**Figure 3:**
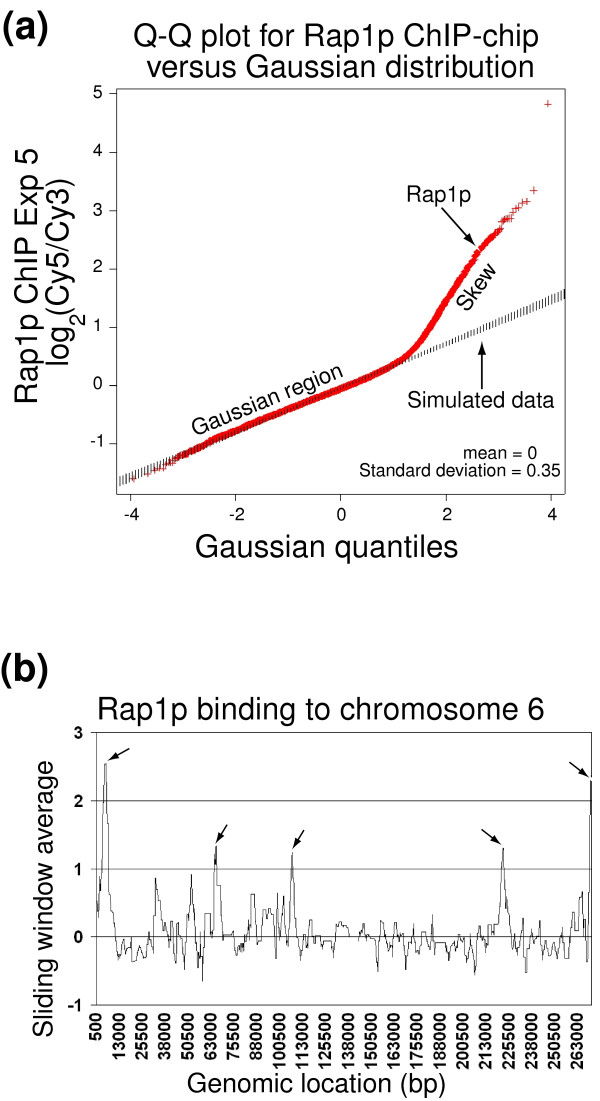
Characteristics of ChIP-chip data. **(a) **A quantile-quantile plot (QQ plot) for one representative Rap1p ChIP-chip experiment (red) against Gaussian distribution with a standard deviation of 0.35 and a mean of 0 (black bars). The upper and lower bounds of the black dashed line represent extreme values for 10,000 simulated Gaussian distributions with the above parameters. For Rap1 about 92% of the data fit the Gaussian distribution. The top 8% is skewed away from the simulated data. **(b) **A sliding window analysis for yeast chromosome VI produced by ChIPOTle for four Rap1p replicates [13]. Window size is 1 kilobase (kb) with 0.25 kb step size. The Rap1p binding sites are identified with arrows.

The second difference in the interpretation of ChIP-chip and traditional gene expression data is that in expression experiments, the data are two-tailed and roughly symmetric. That is, there is biological significance associated with both low and high ratio measurements, and these measurements often occur with similar frequencies. In contrast, the measurements derived from ChIP-chip experiments arise as a mixture of two distributions. The first corresponds to the population of genomic fragments specifically enriched by the ChIP, and the second corresponds to the remaining population of genomic DNA that is not ChIP enriched and therefore represents background, or noise. The observed distribution of the log_2 _ratios is therefore asymmetric about zero, with a distinct, positively oriented skew (Figure 3a). The left-hand side of the distribution (the negative log ratios) is approximately Gaussian, but the positive log ratios exhibit a heavier non-Gaussian tail. For the vast majority of ChIP-chip experiments, the genomic regions of biological interest will be confined to the positive side of the distribution, and the negative log ratios will arise solely from fragments that are considered to be background. Under the additional assumption that the distribution of unenriched fragments is symmetric about zero, we can estimate the distribution of background ratios using only the observed negative log ratios as a guide [6].

The type of microarray used in a ChIP-chip experiment affects how the data can be analyzed. Two array designs are typically used for ChIP-chips: tiled or promoter-specific arrays. Promoter-specific arrays generally contain a single arrayed element to represent each regulatory region of interest. These arrays are valuable when binding is known to be confined to regulatory sequences close to transcriptional start sites of the selected genes [7], but they become less powerful when binding is not as well characterized or is spread over a large genomic area. The other type, namely tiled arrays, are best suited to ChIP-chip. The term 'tiled array', or sometimes 'tiling-path array', refers to arrays containing DNA fragments designed to cover large genomic regions or whole chromosomes with few or no gaps between arrayed elements [8,9]. Tiled arrays are advantageous because they do not require prior knowledge of potential binding targets, and they allow one to utilize the 'neighbor effect' in data analysis.

In this report we describe ChIPOTle (Chromatin ImmunoPrecipitation On Tiled arrays), software created expressly for the analysis of ChIP-chip data obtained using tiled arrays, which allow us to exploit both the 'single-tail' and 'neighbor effect'. ChIPOTle uses a sliding window approach to identify potential sites of enrichment, and then estimates the significance of enrichment for a genomic region using a standard Gaussian error function. ChIPOTle is delivered as a Microsoft Excel macro written in Visual Basic, which should facilitate widespread adoption and provide a platform for custom applications. Before ChIPOTle, to our knowledge the only publicly available program designed expressly for ChIP-chip data analysis was PeakFinder [10]. ChIPOTle offers several improvements, including accurate and powerful *P *value estimation and improved usability. ChIPOTle is available online (Additional data file 1) [11].

## The ChIPOTle algorithm

ChIPOTle first sorts the arrayed elements by genomic location. To find potential areas of ChIP enrichment, a window of user-defined size (default 1 kilobase) is then moved stepwise (user-defined step size; default 0.25 kilobase) along the tiled region. At each step the average log_2 _ratio for the window is calculated by taking the simple average of all ratios reported by arrayed elements that overlap with the window to any degree. The average is unweighted, and therefore it is not dependent on the proportion of the element within the window; it depends only on whether it is present or absent. The window is then moved unidirectionally along the chromosome by the step size and the same calculation is repeated for each distinct window, until the end of the chromosome is reached. The arrayed elements need not be evenly spaced or of equal lengths. ChIPOTle can be used with any genome.

As described in more detail below, the resulting sliding window averages can be represented as a graph, with genomic position on the horizontal axis and average log_2 _ratio on the vertical axis. In this way, genomic binding locations are represented as a series of peaks (Figure 3b). Averaging the log_2 _ratios of elements in a window accounts for the neighbor effect, because the peak generated by a spuriously high signal will be reduced by averaging its value with the ratios of neighboring elements, which are very unlikely also to be high purely by chance.

ChIPOTle assigns a *P *value to the average log ratio within each window, under the null hypothesis that the observed log ratios are independent, identically distributed, and random variables, having a Gaussian distribution with a mean of zero. The variance of the observations is estimated by the average sum of the squared negative log ratios. Under the null hypothesis, the distribution of the average log_2 _ratio within each window is again Gaussian, with mean zero and variance equal to the variance of a single log ratio divided by the number of elements in the window. Thus, the nominal *P *value for a window with average ratio *w *can be calculated using the standard error function (ERF) as follows:



where σ is the standard deviation for the background distribution and n is the number of microarray elements used in the window. The *P *values reported by ChIPOTle are corrected for multiple comparisons using the conservative Bonferroni correction. As an alternative to using a Gaussian distribution for the background, ChIPOTle can estimate the *P *value for a region using a permutation-based approach (Additional data file 2).

## Using ChIPOTle

Detailed instructions for the installation and use of ChIPOTle are available in the read-me file that accompanies the program (Additional data file 2). Once ChIPOTle has been correctly added to the Excel Add-Ins menu or opened manually, a new menu option will appear in the Excel Tools menu. ChIPOTle must be run from an active Excel spreadsheet containing five columns: the name of each arrayed element, chromosome name, start coordinate in base-pairs, end coordinate in base-pairs, and the log_2 _ratio from the ChIP-chip experiment(s). The ratio values supplied to ChIPOTle can be a single measurement from a single experiment or an average, weighted average, or median of ratio values calculated from multiple replicates. When using data from multiple replicates, before combining the data each array must be appropriately normalized to remove systematic nonbiological effects that might otherwise influence the results [1]. For single channel experiments, pseudo-ratios must be created before using ChIPOTle. Pseudo-ratios may be created by dividing the intensity value at each arrayed element by the median intensity value for all arrayed elements.

Through a dialog window, ChIPOTle will ask for the location of each data column. The user will also be prompted to provide the window size, step size, and the desired technique for determining peak significance. For the latter parameter, the user can choose (1) a simple peak height cutoff; (2) assume a Gaussian background distribution for calculation of window average *P *values; or (3) estimate the background distribution for calculation of window average *P *values via a permutation-based simulation. If option 1 is selected then the user is prompted to enter the peak height; for option 2 the user is prompted to provide the significance *P *value cutoff; and for option 3 the user is prompted to provide the number of simulations and the significance *P *value cutoff to be used in the permutation analysis. Any region with a *P *value lower then the selected cutoff will be recorded and summarized in the "Significant Regions" and "Peaks" worksheets.

## Parameter optimization

As described above, ChIPOTle has three important user-defined parameters: *P *value cutoff, window size, and step size. These parameters will affect the output, and can be adjusted according to the experiment and the array design. The *P *value cutoff should be set at a level that produces a false discovery rate with which the user is comfortable. The "Significant Negative Regions" sheet provides an empirical estimate of the number of false-positive findings for the selected *P *value cutoff, and so the user can use this information to estimate the false-positive rate and adjust the *P *value cutoff (see below). The numbers of acceptable false-positive and false-negative findings will vary depending on the goals of the study.

The next parameter to set is window size. Ideally, for a given protein-DNA interaction, one would like to capture the maximal amount of ChIP signal associated with a single binding event, and none of the noise, in a single window. Therefore, in most ChIP-chip experiments the window size should be adjusted to approximately the average shear size of the chromatin. The average shear size is suggested because the size of the window must be balanced against making it so large that noise from adjacent genomic regions is included in the measurement, and against making the window so small that data from adjacent spots is excluded, diminishing the power of windowing to utilize the neighbor effect. Although this parameter is largely independent of array platform or array resolution, slightly smaller windows may be more effective on higher resolution arrays.

Optimization of step size depends on both the array resolution and the window size. The step size should be adjusted such that it is less than half of the array resolution, with array resolution defined as the distance between the start of one arrayed element and the start of the next. Thereby, the measurement recorded at each arrayed element will be used in the calculation of at least three windows, ensuring that every arrayed element has the opportunity to be centered under a peak. Window size is also an important factor because some overlap of windows is desirable in order to detect peaks at unknown locations. Taken together, we suggest setting the step size to the maximum value that is both less than half of the array resolution and less than or equal to one-quarter of the window size. For very high-resolution arrays (less than about 50 base-pairs), step sizes smaller than the array resolution may not improve results.

## ChIPOTle output

ChIPOTle creates several output sheets with the following names: SummarySheet, Significant Regions, Significant Negative Regions, Chromosomes aveP, Peaks, and Description. The SummarySheet contains all the input data used to run ChIPOTle, now sorted by chromosome and start coordinate. For each window that meets the significance criteria specified by the user, the Significant Regions sheet contains the following: chromosome assignment, center coordinate, number of independent arrayed elements within each window, and names of the arrayed elements that comprise the window. Significant Negative Regions is similar to Significant Regions, but instead it contains all of the windows that meet the significance criteria but are sign-flipped. The number of windows reported in this sheet can be used as an estimate of the number of false-positive findings expected for the selected or estimated cutoff. Chromosome aveP contains the names of the arrayed elements that comprise each window, and the chromosome, center coordinate, and value of all windows, regardless of whether they meet the significance criterion. The values from this sheet, for example, were used to make Figure 3b.

The data written to the "Peaks" sheet are similar to those reported in "Significant Regions", except that all neighboring windows meeting the significance criteria are collapsed into a single peak. Therefore, a peak is defined as any window with a *P *value that meets the significance criterion defined by the user and all neighboring windows that also meet the significance criteria. In this sheet, each peak is listed in order of its occurrence along the chromosome, along with the highest window for each peak, highest raw log_2 _ratio for any element within the peak, start coordinate of the peak, the width of portion of the peak above the significance cutoff, 'array density' of the peak, and the *P *value for that peak. The array density value is defined as the average number of arrayed elements used to calculate the window values for all windows that comprise the peak. Therefore, the array density value provides an estimate of the number of actual raw data measurements that underlie each peak.

The last sheet, Description, contains a summary of the ChIPOTle execution parameters, which include the date and time, the selected window size, the step size, the significance method chosen and corresponding parameters, the number of significantly enriched peaks, and the total number of windows.

## Properties of ChIP-chip data

A plot of the sliding window values generated by ChIPOTle for a Rap1p ChIP-chip reveals two important characteristics of this type of data (Figure 3b). The first is an absence of deep negative peaks. In ChIP-chip experiments, negative log ratios are not caused by specific depletion of genomic fragments but by noise. Therefore, after averaging with neighboring genomic elements, their window average will tend to be small. The second is the presence of tall positive peaks that extend well above background.

## Comparing ChIPOTle with other techniques used to analyze ChIP-chip data

**Figure 4:**
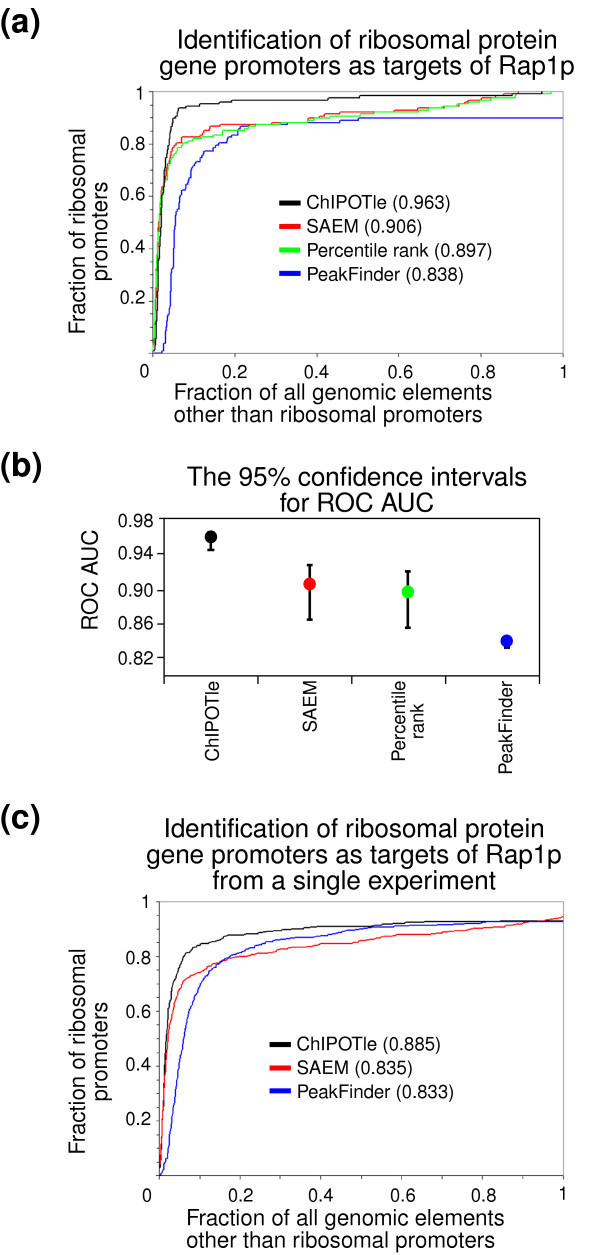
Comparison of ChIPOTle with other ChIP-chip analysis approaches. **(a) **ChIPOTle, the single-array error model (SAEM), median percentile rank, and PeakFinder were used to analyze the same four Rap1p ChIP-chip replicates reported by Lieb and coworkers [13], and judged by their ability to determine enrichment of ribosomal protein gene (RPG) promoters. The binding site for Rap1p is found in most (>90%) RPG promoters [14], which represent approximately half of Rap1p's total *in vivo *targets. Receiver operating characteristic (ROC) curves summarize the power of each technique and are equivalent to a plot of the true-positive rate (fraction of ribosomal promoters) versus the false-positive rate (fraction of all genomic elements other than ribosomal promoters). Each technique is judged by means of the area under the ROC curve (AUC). An AUC value of 0.5, corresponding to a diagonal ROC curve, is expected by chance, whereas a value of 1.0 indicates a technique that predicts targets perfectly. ChIPOTle (AUC = 0.963) outperformed the other techniques tested here (SAEM: AUC = 0.906; median percentile rank: AUC = 0.897; and PeakFinder: AUC = 0.823). When comparing ChIPOTle with PeakFinder, we used the default settings for smoothing (n = 5 [11-point] smoothing with 7 rounds). In addition, we attempted to optimize the settings by trying varying levels of smoothing, including 7-point and 13-point, which produced similar results. Rap1p's strongest binding sites are located at the telomeres, which are not included with our defined 'true positive' set of RPG promoters. Therefore, the false-positive rate will be somewhat inflated, which will decrease the AUC for all techniques. This is reflected in the ROC curves by the low true-positive rate at the extreme left of the plot. **(b) **The 95% confidence interval for the AUC for each analysis technique was estimated by bootstrap resampling of RPG occurrence and enrichment value (1,000 iterations) as measured in each technique (*P *value, percentile rank, or ySmooth). Boostrapping of raw data was not practical because of inability to automate all four analysis methods. **(c) **ROC curves comparing ChIPOTle, SAEM, and PeakFinder with respect to their ability to identify enrichment of RPG promoters from a single experiment. The average true-positive rate (fraction of ribosomal promoters) versus false-positive rate (fraction of all genomic elements other than ribosomal promoters) for the four individual experiments is plotted. The three techniques performed extremely well, but ChIPOTle (AUC = 0.885) outperformed both SAEM (AUC = 0.835) and PeakFinder (AUC = 0.833).

We compared ChIPOTle with three other analysis techniques commonly used to analyze ChIP-chip experiments: the single array error model (SAEM) [6,7,12], percentile rank analysis [13], and PeakFinder (smoothing settings: n = 5, rounds = 7) [10]. All four techniques were used to analyze four biological replicates (experiments 5, 6, 8, and 9) from the Rap1p binding dataset in yeast reported by Lieb and coworkers [13]. To compare the power of the four techniques quantitatively, they were judged by their ability to identify the 127 promoters of the ribosomal protein genes (RPGs) as targets of Rap1p binding. As a group, these promoters are known targets of Rap1p, and almost all contain consensus Rap1p-binding sites [14]. By using this functionally defined set, we avoided using any particular ChIP dataset to define our 'gold standard'. The targets identified by each technique were sorted by *P *value (ChIPOTle and SAEM), median percentile rank (percentile rank), or ySmooth value (PeakFinder). We then used receiver operator characteristic (ROC) plots to show how true positives (sensitivity) were captured in relation to false positives (specificity) for all values output by each method (Figure 4a). The power of each technique was then quantitated as the area under the ROC curve (AUC). An analysis technique that selected targets randomly would have an AUC of about 0.5; higher values are better (maximum = 1).

In using the Rap1p ChIP-chip data to identify the promoters of RPGs, all of the techniques worked well, but ChIPOTle (Figure 4a, black line; AUC = 0.963) performed considerably better then the other techniques (SAEM: AUC = 0.906, percentile rank AUC = 0.897; PeakFinder: AUC = 0.838). The 95% confidence interval for each AUC value (Figure 4b) was estimated by bootstrap resampling of RPG occurrence and enrichment value as measured in each technique (*P *value, percentile rank, or ySmooth) [15].

We next compared the ability of ChIPOTle, SAEM, and PeakFinder to identify accurately the RPG promoters from a ChIP-chip hybridization to a single microarray. This analysis cannot be performed with the percentile rank analysis because this technique requires experimental replicates. We analyzed each individual experiment independently and determined the average true-positive rate versus the false-positive rate (Figure 4c). All three techniques performed extremely well, but ChIPOTle (AUC = 0.885) outperformed both SAEM (AUC = 0.835) and PeakFinder (AUC = 0.833). In addition, ChIPOTle produced higher AUC values than both SAEM and PeakFinder for each individual experiment (data not shown).

## Discussion

ChIPOTle is a Microsoft Excel macro that is designed for use in the analysis of data from ChIP-chip experiments. ChIPOTle exploits the unique characteristics of ChIP-chip data, including enrichment of DNA genomically adjacent to sites of protein-DNA interaction, and the single-tailed nature of the data, to define peaks of enrichment and their significance. ChIPOTle is very quick and easy to use. The user is prompted to select the five columns containing their data and the significance technique to be used. The program then returns the genomic regions that were enriched by the ChIP according to the data and the specified statistical parameters. In its current implementation, ChIPOTle is restricted in functionality by the limitations of Excel worksheets to 65,536 rows by 256 columns. Therefore, if the dataset of interest is derived from an array containing more then 65,536 unique elements or if the total number of windows generated exceeds 5.5 million, then the data will have to be separated into subsets (for example, individual chromosomes) if they are to be analyzed using ChIPOTle.

As currently implemented, the significance analysis in ChIPOTle is carried out under the assumption that the log_2 _ratios of the arrayed elements are independent and Gaussian distributed, with mean zero and common variance. Under this assumption, a nominal *P *value may be assigned to each window using the standard Gaussian cumulative distribution function, or an appropriate bound having closed form. Multiple comparisons can then be addressed via a Bonferroni correction or through an estimated false-discovery rate. In either case, the tail behavior of the Gaussian distribution will have a strong effect on the corrected *P *values.

As a more conservative alternative to the Gaussian approach, one could derive nominal *P *values from each window using a null distribution with heavier tails than the Gaussian. A natural choice, consistent with the observed histogram of log_2 _ratios, is a t-type distribution. Formally, one may adopt the null hypothesis that the observed log_2 _ratios are independent and distributed as cT, where c is a positive scaling factor and T has a standard t distribution with v degrees of freedom. In order to obtain nominal *P *values, one then needs estimates of c and v, and bounds on the probability that a sum of independent t-distributed random variables exceeds a threshold. Estimates of c and v can be obtained through moment-based methods. Suitable probability bounds with good small-sample properties are currently under investigation.

ChIPOTle, while using novel approaches, identifies a set of sites similar to that defined by other techniques (PeakFinder, SAEM, and percentile rank analysis) used for analysis of data from ChIP-chip experiments. However, the use of a sliding window allows ChIPOTle to identify enriched regions more accurately, especially after only one experiment. This is useful because when one is performing a ChIP-chip experiment for the first time with a new protein or antibody, it is often difficult to determine whether the ChIP was successful, especially for a protein with an undefined binding pattern. The ability to determine binding sites correctly using fewer replicates will be very important for larger, more complex genomes. Complete high-density tiled arrays for mammalian genomes require many arrays for each experiment, meaning that performing the ideal number of replicates can be prohibitively expensive. In mammalian systems, instead of performing all of the replicates of a ChIP-chip experiment on whole-genome arrays, preliminary experiments using whole-genome arrays can be used to find likely targets. Once these likely targets are identified, the array could be redesigned to include all prospective targets and appropriate controls on a single array. In addition to its utility as a general ChIP-chip analysis tool, ChIPOTle will make prescreening more accurate and will enhance the power and accuracy of this approach.

## Additional data files

The following additional files are included with the online version of this paper: The Excel Add-In ChIPOTle v 1.0 (Additional data file 1), a pdf file containing detailed instructions for the installation and use of ChIPOTle (Additional data file 2), and an Excel file containing the Rap1p binding data used to make the comparisons between the different techniques (Additional data file 3).

## Supplementary Material

Additional data file 1 An Excel Add-In ChIPOTle v 1.0. Click here for file

Additional data file 2 A PDF file containing detailed instructions for the installation and use of ChIPOTle. Click here for file

Additional data file 3 An Excel file containing the Rap1p binding data used to make the comparisons between the different techniques. Click here for file
