# Supplementary material for: ChIPOTle: a user-friendly tool for the analysis of ChIP-chip data
Source: Genome Biol. 2005 Oct 19;6(11):R97. doi: 10.1186/gb-2005-6-11-r97 (PMC1297653; doi:10.1186/gb-2005-6-11-r97)

ChIPOTle v1.0: A Tool to Identify Genomic Regions Enriched in ChIP-chip Experiments

Michael Buck, UNC-CH Lieb lab, 2005
mjbuck@unc.email.edu

Description
ChIPOTle is a Microsoft Excel add-in Macro that analyzes yeast ChIP-chip data generated on whole-genome tiled arrays.

Introduction
In contrast to mRNA microarray experiments, in which each arrayed element usually measures the abundance of one mRNA species, in ChIP-chip experiments each element measures the abundance of a population of fragments of assorted lengths due to chromatin shearing. Therefore, arrayed elements representing genomic regions 1- to 2-kb downstream or upstream from the binding site will also detect enrichment. This effect produces a peak over several arrayed elements containing genomically adjacent DNA. This is non-random behavior that is not expected from spuriously high ratio measurements. ChIPOTle takes advantage of this fact and uses it as an independent confirmation of enrichment for a given genomic region.

ChIPOTle works by determining a sliding window average across each chromosome. A window of selected size (default 1 kb) is slid across a region or chromosome, and the average $\log_2$ ratio of any arrayed elements that fall within that window is determined. The window is moved downstream by the step size (default 0.25 kb), and then the calculation is repeated iteratively for the whole chromosome. This sliding average will identify binding sites as peaks. The height of peaks caused by spuriously high ratios will be reduced, since the probability of a neighboring genomic element also having a high ratio is low. ChIPOTle also defines a array density value for each peak based on the number of independent arrayed elements used to construct the peak.

The utility of this approach is that it does not depend on the absolute number of targets, but on the density of their distribution. It is appropriate for detecting any number of targets that are distributed with a frequency less than approximately three times the average sheared chromatin size. For example, if the average sheared chromatin size were 1 kb, this method would be useful for the detection of any protein predicted to be spaced at intervals of at least 3 kb. A drawback to this approach is that it requires high-resolution tiling arrays.

Setting up and running the Microsoft Excel CHIPOTLE Macro
1) Start excel by double clicking on the ChIPOTle.xla file. You may get a security warning, and if so click "enable marcos". This will start Excel with the macro loaded. ChIPOTle will add a menu option to the excel Tools toolbar “CHIPOTLE”.

> *If you are having problems, make sure Excel’s security setting for macros is set to medium or low. Excel’s security setting may be changed in the tools-macros -security menu option.*

2) Open a spreadsheet containing your data in five columns (Spot name, $\log_2$ ratio, Chromosome or region ID, start coordinate, and stop coordinate).

3) To run ChIPOTle , go to the tools – CHIPOTLE menu option. You will be presented with a set of options.

Setting Parameters

1.) You will be prompted to select the cells containing the required input data. Select the cells containing the spot names (string), log ratios (real), chromosome number (string or number), start and stop coordinate (integer).

2) Selecting window size and step size: The program was designed to use a window size equal to the average shearing size of the DNA used in the ChIP. The step size should be set at ~¼ the shear size. Default settings – window size 1000 bases, step size 250 bases (Figure 1) .

3) Select significance criteria: (A) Peak height cutoff (log_2 ratio value, default 1.0), to use as a cutoff for significant peaks. (B) Assume that the background distribution is Gaussian. (C) Estimate the background distribution using a permutation simulation. See "Picking a significance criterion" below for more details.

4) Permutation Parameters – If you selected permutation simulation, two additional parameters are required before the program will run. These are the number of permutations and the p-value. The number of permutations is the number of times all the data will be shuffled and the sliding window used to determine the negative peak distribution. The larger this number the longer it takes to run the program. The p-value is used in determining the cutoff via permutation simulations. In addition, the user should pay close attention to the number of significant negative regions (Significant Negative Regions). If there are many significant negative regions when compared to significant positive regions (Significant Regions), then the p-value cutoff should be decreased. A p-value cutoff that produces about 50 times more significant regions then false regions may be satisfactory.

Running the program

1) ChIPOTle retrieves the chromosome number, start, and end coordinates for each array element from the inputted data.

2) If selected, ChIPOTle estimates a cutoff for the selected p-value. The program updates its progress in the bottom left of the window.

3) The program calculates the sliding-window average for your data and outputs several data sheets.

Output

1) ChIPOTle will add the following sheets to the data workbook:

SummarySheet	- Contains all the data with the spot start and stop
Significant Regions	- Lists all regions above the positive cutoff
Significant Negative Regions	- Lists all regions below the negative cutoff
Chromosomes aveP	- Contains full output for each chromosome
Peaks	- Lists all the positive peaks above the positive cutoff
Description	- Lists the settings for CHIPOTLE run
FDR	- Lists all peaks identified by CHIPOTLE with the p-value and q-value for false discovery rate when using the permutation simulation approach.

2) Output Column Labels:

A) “Significant Regions” and “Significant Negative Regions”

Chromosome – Chromosome Number
Position – Start of window
Ave Log Ratio – Sliding window average for that region starting at position
# of spots – Counts the number of independent spots used to get the average
Names – List the name of the spots for that region

B) Peaks above cutoff

Peak Number – Peak ID number by location
High Average – Highest window average for that peak
High Ratio – Highest log ratio for that peak
High Spot – The array element with the highest log ratio
Length – The length of the peak above the cutoff
Chromosome – Chromosome location or region
Peak Start – The first window average above the cutoff
Array density – A measure of the number of independent spots in the peak. A “1” means that only one spot was used it make that peak above the cutoff, therefore, this peak may not be reliably enriched.
P-value – Probability of enrichment via Gaussian or permutation

C) FDR

Peak Number – Peak ID number by height
High Average – Highest window average for that peak
High Ratio – Highest log ratio for that peak
High Spot – The array element with the highest log ratio
Chromosome – Chromosome location or region
Peak Start – The first window average above the cutoff
P-value – Probability of enrichment via permutation
Q-value – Q-value for determining FDR

Figure 1. Loading required input data and running ChIPOTle

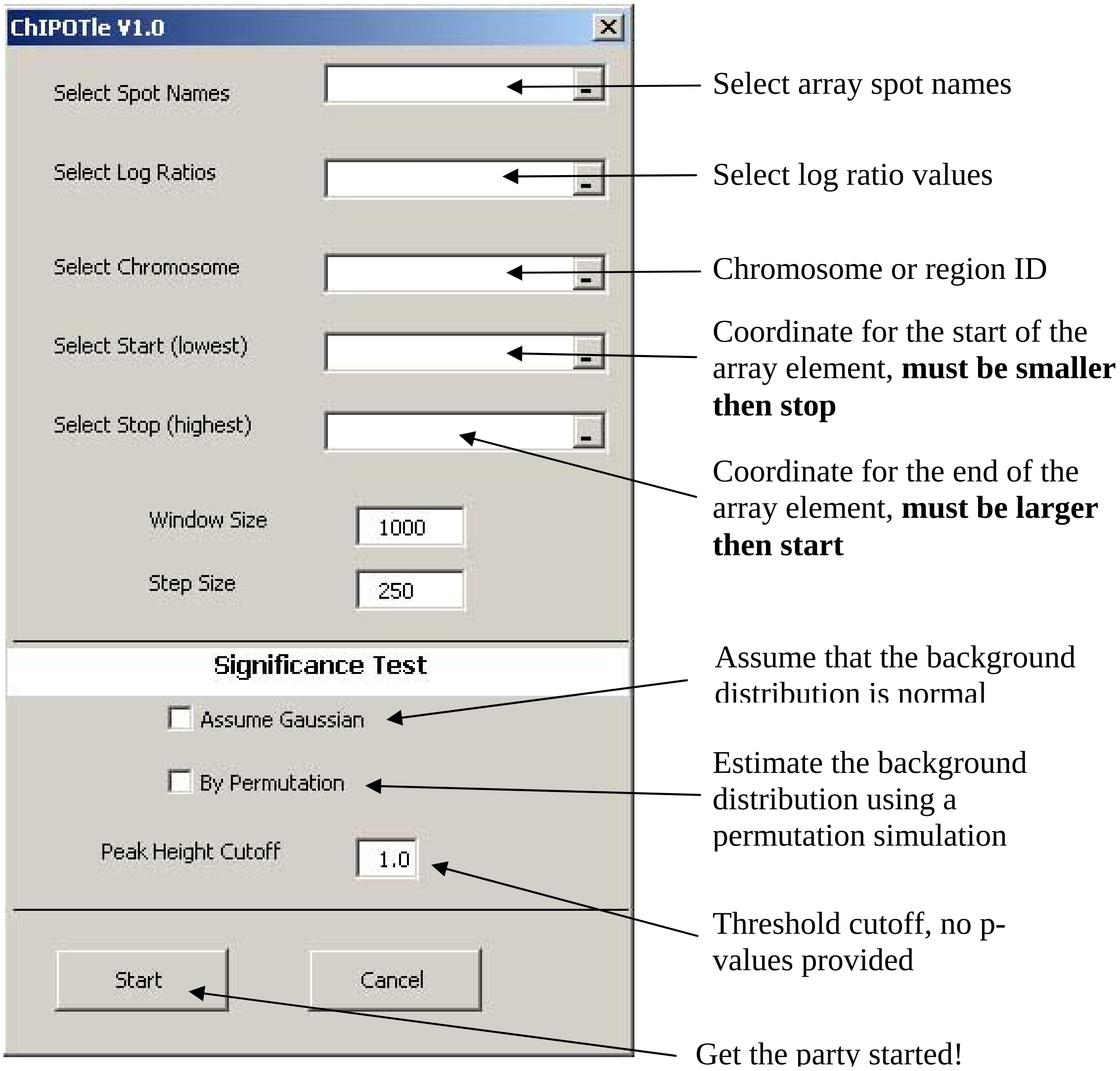

Figure 2. Looking at Results

SHEET – “Peaks above cutoff”

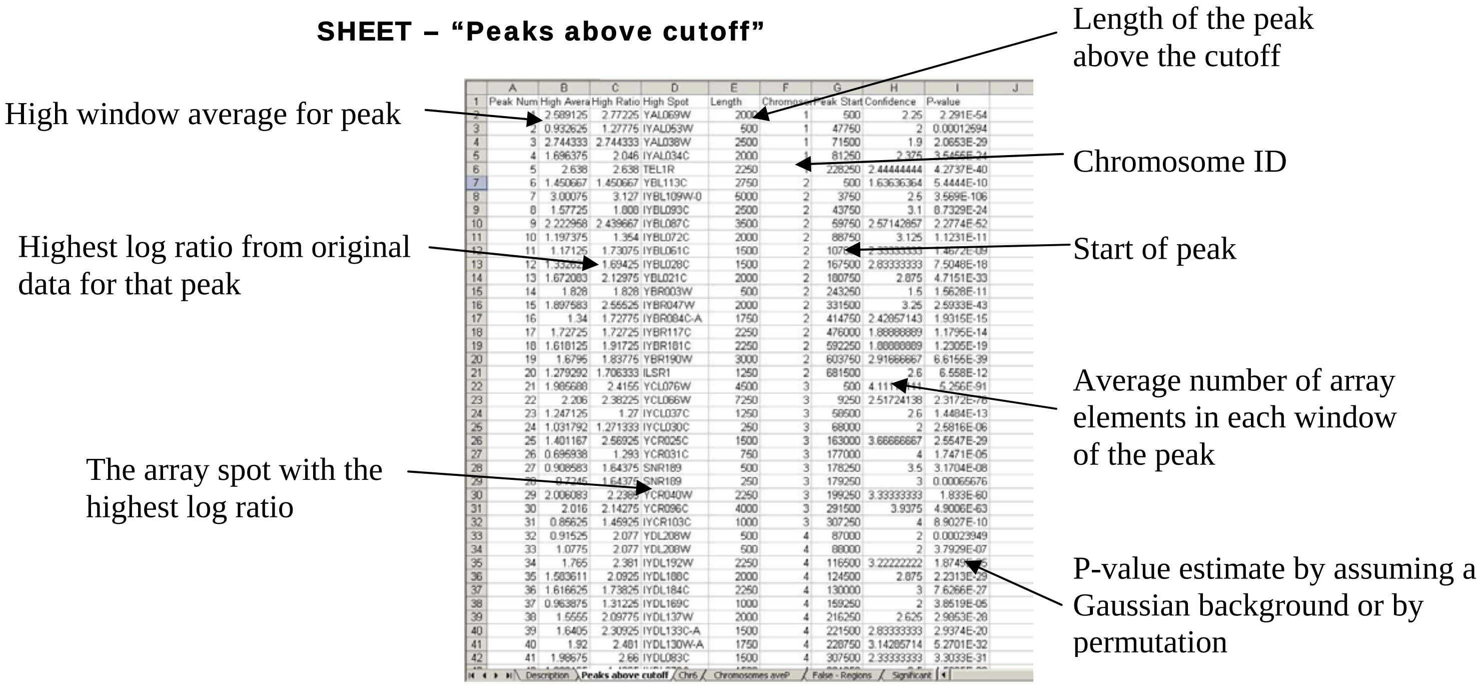

	A	B	C	D	E	F	G	H	I	J
1	Peak Num	High Avera	High Ratio	High Spot	Length	Chromoso	Peak Start	Confidence	P-value	
2	[illegible]	2.589125	2.77225	YAL069W	[illegible]	1	500	2.25	2.291E-54	
3	2	0.932625	1.27775	IYAL053W	500	1	47750	2	0.00012594	
4	3	2.744333	2.744333	YAL038W	2500	1	71500	1.9	2.0653E-29	
5	4	1.696375	2.046	IYAL034C	2000	[illegible]	81250	2.375	3.5456E-24	
6	5	2.638	2.638	TEL1R	2250	[illegible]	228250	2.44444444	4.2737E-40	
7	6	1.450667	1.450667	YBL113C	2750	2	500	1.63636364	5.4444E-10	
8	7	3.00075	3.127	IYBL109W-0	5000	2	3750	2.5	3.569E-106	
9	8	1.57725	1.808	IYBL093C	2500	2	43750	3.1	8.7329E-24	
10	9	2.222958	2.439667	IYBL087C	3500	2	59750	2.57142857	2.2774E-52	
11	10	1.197375	1.354	IYBL072C	2000	2	88750	3.125	1.1231E-11	
12	11	1.17125	1.73075	IYBL061C	1500	2	[illegible]	[illegible]	[illegible]	
13	12	[illegible]	1.69425	IYBL028C	1500	2	167500	2.83333333	7.5048E-18	
14	13	1.672083	2.12975	YBL021C	2000	2	180750	2.875	4.7151E-33	
15	14	1.828	1.828	YBR003W	500	2	243250	1.5	1.5628E-11	
16	15	1.897583	2.55525	IYBR047W	2000	2	331500	3.25	2.5933E-43	
17	16	1.34	1.72775	IYBR084C-A	1750	2	414750	2.42857143	1.9315E-15	
18	17	1.72725	1.72725	IYBR117C	2250	2	476000	1.88888889	1.1795E-14	
19	18	1.618125	1.91725	IYBR181C	2250	2	592250	1.88888889	1.2305E-19	
20	19	1.6795	1.83775	YBR190W	3000	2	603750	2.91666667	6.6155E-39	
21	20	1.279292	1.706333	ILSR1	1250	2	681500	2.6	6.558E-12	
22	21	1.985688	2.4155	YCL076W	4500	3	500	[illegible]	5.256E-91	
23	22	2.206	2.38225	YCL066W	7250	3	9250	2.51724138	2.3172E-76	
24	23	1.247125	1.27	IYCL037C	1250	3	58500	2.6	1.4484E-13	
25	24	1.031792	1.271333	IYCL030C	250	3	68000	2	2.5816E-06	
26	25	1.401167	2.56925	YCR025C	1500	3	163000	3.66666667	2.5547E-29	
27	26	0.696938	1.293	YCR031C	750	3	177000	4	1.7471E-06	
28	27	0.908583	1.64375	SNR189	500	3	178250	3.5	3.1704E-08	
29	[illegible]	[illegible]	[illegible]	SNR189	250	3	179250	3	0.00065676	
30	29	2.006083	[illegible]	YCR040W	2250	3	199250	3.33333333	1.833E-60	
31	30	2.016	2.14275	YCR096C	4000	3	291500	3.9375	4.9006E-63	
32	31	0.85625	1.45925	IYCR103C	1000	3	307250	4	8.9027E-10	
33	32	0.91525	2.077	YDL208W	500	4	87000	2	0.00023949	
34	33	1.0775	2.077	YDL208W	500	4	88000	2	3.7929E-07	
35	34	1.765	2.381	IYDL192W	2250	4	116500	3.22222222	[illegible]	
36	35	1.583611	2.0925	IYDL188C	2000	4	124500	2.875	2.2313E-29	
37	36	1.616625	1.73825	IYDL184C	2250	4	130000	3	7.6266E-27	
38	37	0.963875	1.31225	IYDL169C	1000	4	159250	2	3.8519E-05	
39	38	1.5555	2.09775	IYDL137W	2000	4	216250	2.625	2.9853E-28	
40	39	1.6405	2.30925	IYDL133C-A	1500	4	221500	2.83333333	2.9374E-20	
41	40	1.92	2.481	IYDL130W-A	1750	4	228750	3.14285714	5.2701E-32	
42	41	1.98675	2.66	IYDL083C	1500	4	307500	2.33333333	3.3033E-31	

SHEET – “Description”

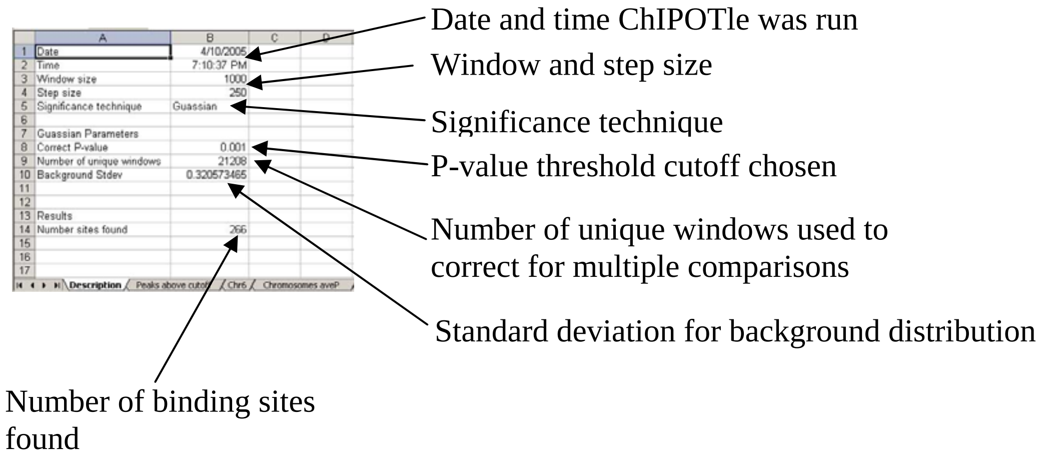

	A	B	C	D
1	Date	4/10/2005		
2	Time	7:10:37 PM		
3	Window size	1000		
4	Step size	250		
5	Significance technique	Guassian		
6				
7	Guassian Parameters			
8	Correct P-value	0.001		
9	Number of unique windows	21208		
10	Background Stdev	0.320573465		
11				
12				
13	Results			
14	Number sites found	266		
15				
16				
17				

Figure 3. Making Charts

Chromosomal Maps of sliding window average - Sheet "Chromosomes aveP" contains all the sliding window average data.

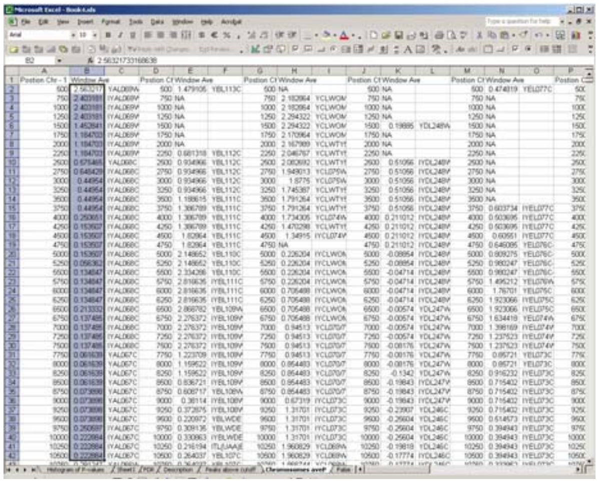

Step 1) Select the window average for the chromosome or region desired.

Step 2) Insert line chart

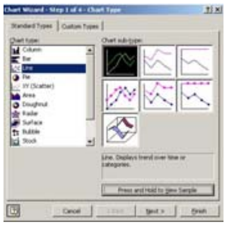

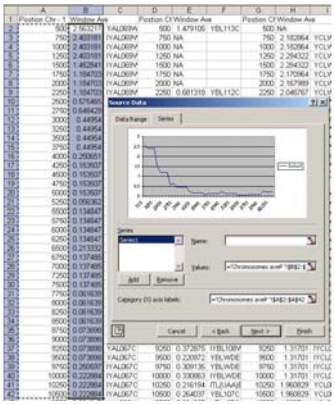

Step 3) Select chromosomal location for category X-axis

Step 4) Label chart as desired

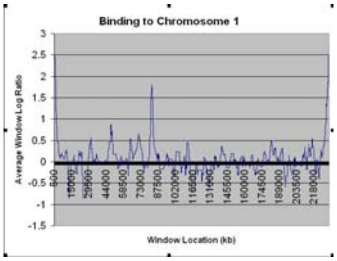

Figure 4. P-value vs Average Window log ratio chart (only for permutation simulations)– Sheet "P-value Histogram" contains the results from the permutation simulation.

Step 1) Select Average log ratio and p-value (cols A and B)

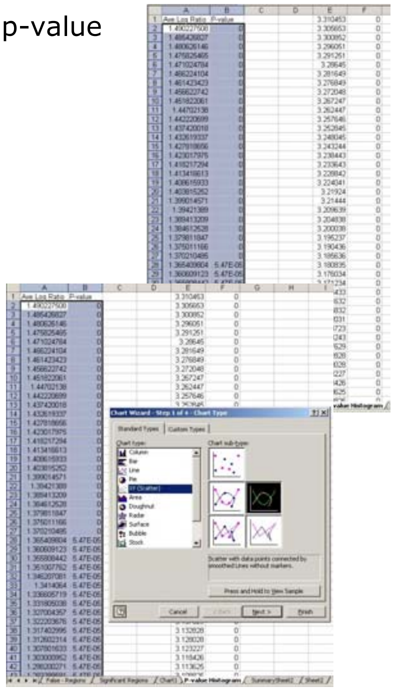

Step 2) Insert xy scatter chart

Step 3) Label chart as desired

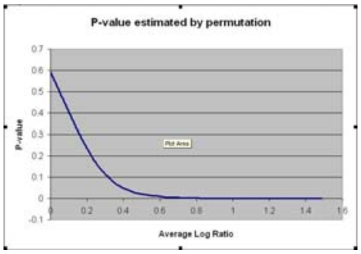

Figure 5. Distribution of all peaks – Sheet "P-value Histogram" contains the height of all peaks found in the experiment.

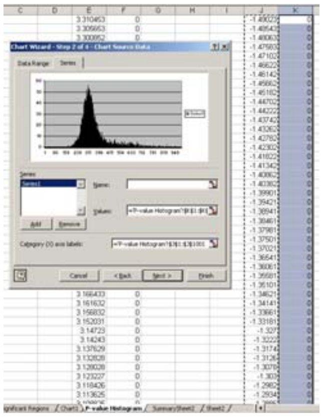

Step 1) Select column K

Step 2) Insert column chart

Step 3) Select column J for category X-axis

Step 4) Label chart as desired

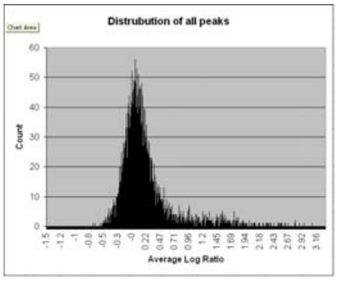

Picking a significance criterion

ChIPOTle has three options for determining the significance of enrichment found in ChIP-chip experiment.

1. Peak height cutoff
   Any peak with a height above the average $\log_2$ ratio inputted will be saved in the Significant Regions and the Peaks worksheets. This approach does not estimate the p-value for each window or peak.

2. Background Gaussian distribution
   The background or non-enriched population is assumed to a symmetric Gaussian distribution about the mean of zero. For most ChIP-chip datasets this is the case but is not true for all experiments. See "Is my data Gaussian" below if your not sure if you data fits this assumption. Using the Gaussian distribution is the most powerful approach in ChIPOTle for estimating the p-value of enrichment. Under the null hypothesis, the distribution of the average $\log_2$ ratio within each window is again Gaussian, with mean zero and variance equal to the variance of a single log ratio divided by the number of elements in the window. Thus the nominal p-value for a window with average ratio *w* can be calculated using the standard error function (ERF) as follows:

   $$(1) \qquad P_{window} = 1 - ERF\left(\bar{w} \big/ \sigma/\sqrt{n} \right)$$

   where σ is the standard deviation for the background distribution, and *n* is the number of microarray elements used in the window. The p-values reported by ChIPOTle are corrected for multiple comparisons using the conservative Bonferroni correction.

3. Estimate background using permutation
   The background or non-enriched population is assumed only to be symmetric about the mean of zero. This approach only looks for peaks in the sliding window averages and does not estimate a p-value for every window. In addition the p-values for peaks are not correct for multiple testing. Therefore, ChIPOTle includes an additional output sheet FDR which contains the false discovery rate statistics. The peaks are identified from the data as any window or group of windows with the same value having a preceding and following window of a lower value. Only these peaks will be tested for enrichment, reducing the total number of statistical test required. The significance of enrichment for a peak is estimated by comparing it's height to the height of peaks caused by chance (non-enriched). The height of peaks caused by chance is estimated by a permutation simulation of all non-enriched regions. Since, ChIP-chip experiments do not specifically deplete any genomic fragments, any array element or peak with negative log ratio can be assumed to belong to the non-enriched population. With the assumption of symmetry about the mean for the non-enriched population we can estimate the complete non-enriched population by reflecting the negative distribution onto the positive axis. For example, a negative peak of depth -0.5, which should occur only by chance, will occur as often as a positive peak of height 0.5 by chance. From this distribution of the non-enriched positive

peaks, CHIPOTLE estimates the probability of enrichment for each peak found.

First the genomic order of the data is randomized and then a sliding window average is determined with the user specifications. Negative peaks are determined and their depth's counted. This is repeated a selected (default = 100) number of times and the distribution of the peaks is used to determine the p-value for enrichment.

Is my data Gaussian?

The quick and easy check using Microsoft excel data "Analysis ToolPak". The steps below demonstrate how to make a plot similar to a Q-Q plot in microsoft excel.

1. Count the number of elements in your dataset.
2. Create a list of random number from a Normal distribution using data analysis toolpak addin.

Figure 6. Creating random Gaussian data

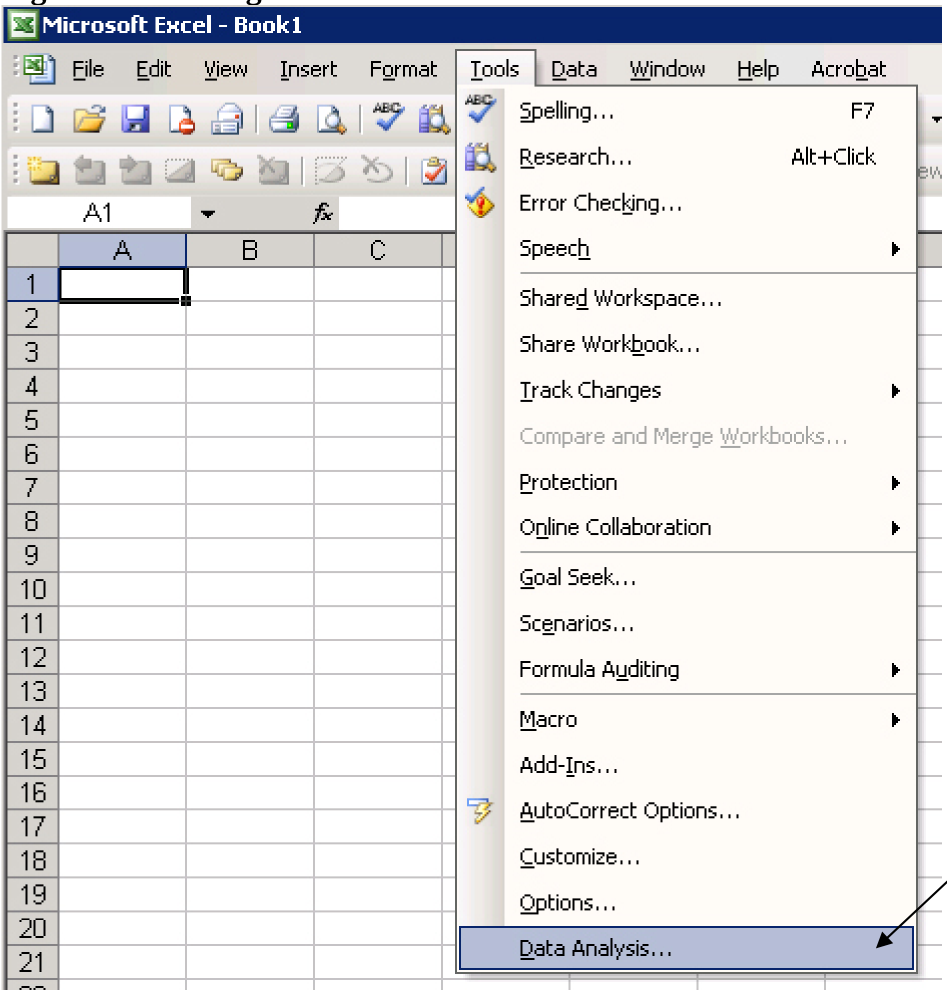

Select data analysis, if you don't have this option make sure you have the ToolPak add-in installed.

Figure 7. Creating random Gaussian data part 2

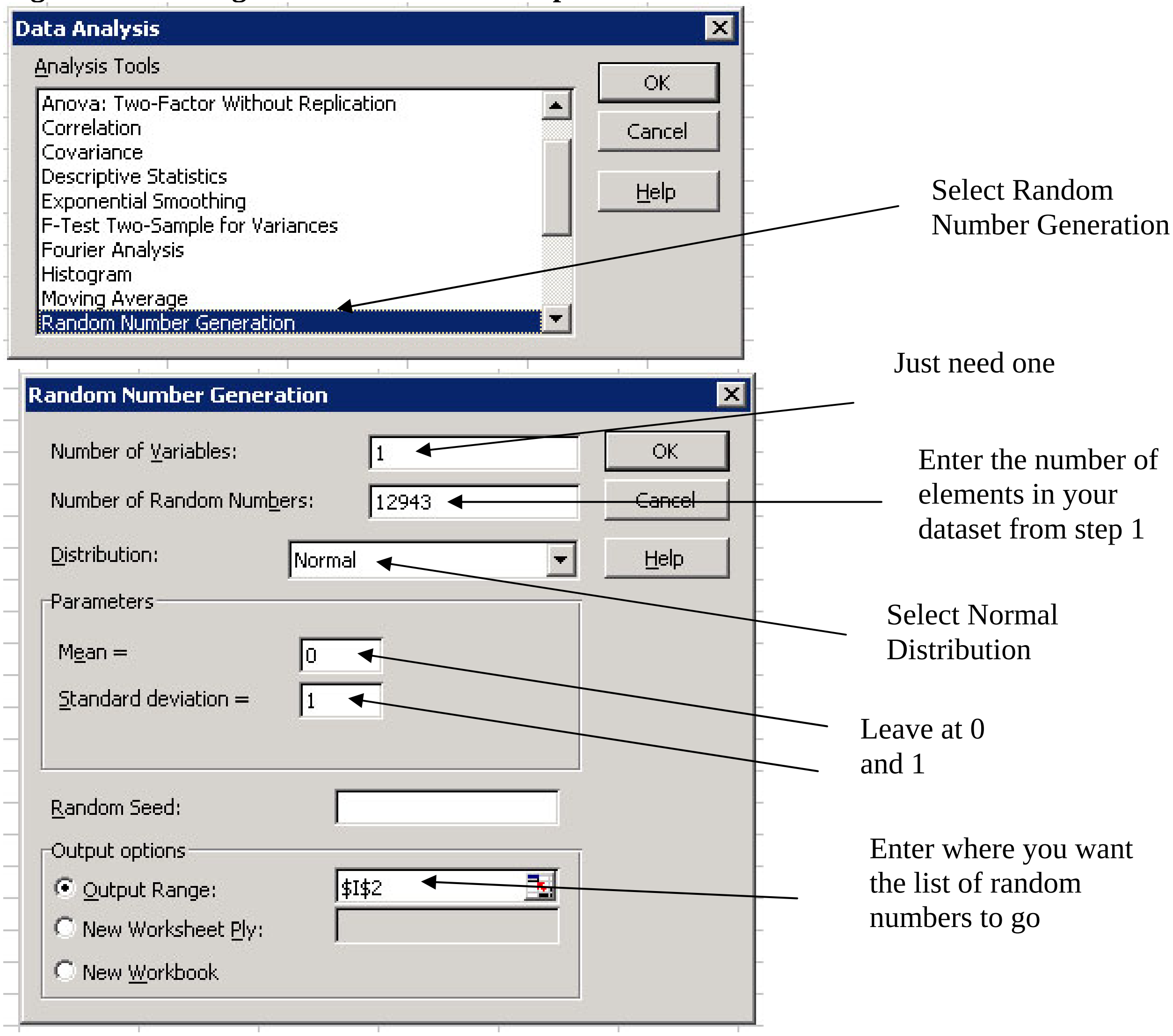

3. Sort your dataset and the random number dataset individually in ascending order

Figure 8. Sorting a list

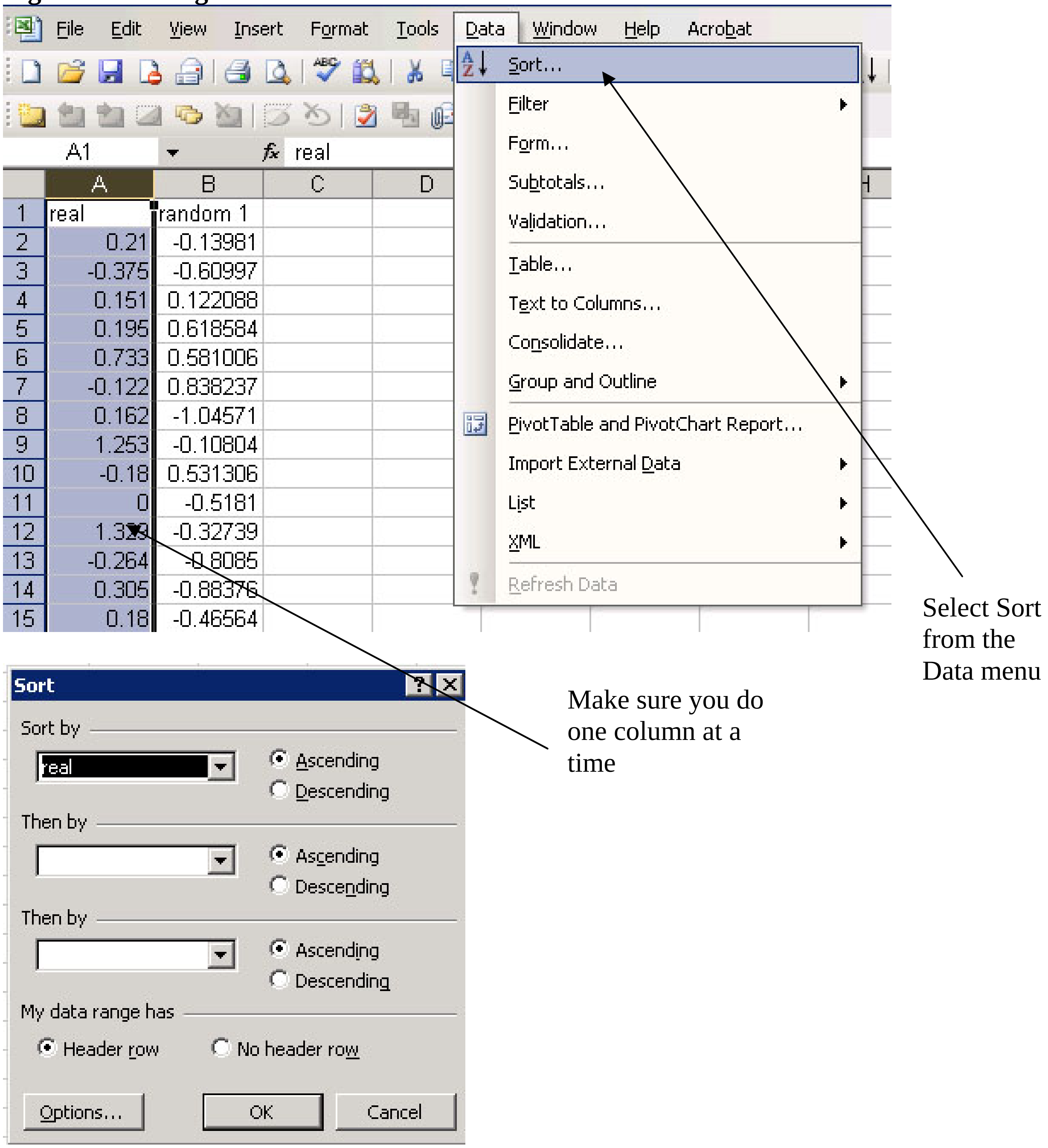

4. Make a x-y plot of the two data sets

Figure 9. Making X-Y plot

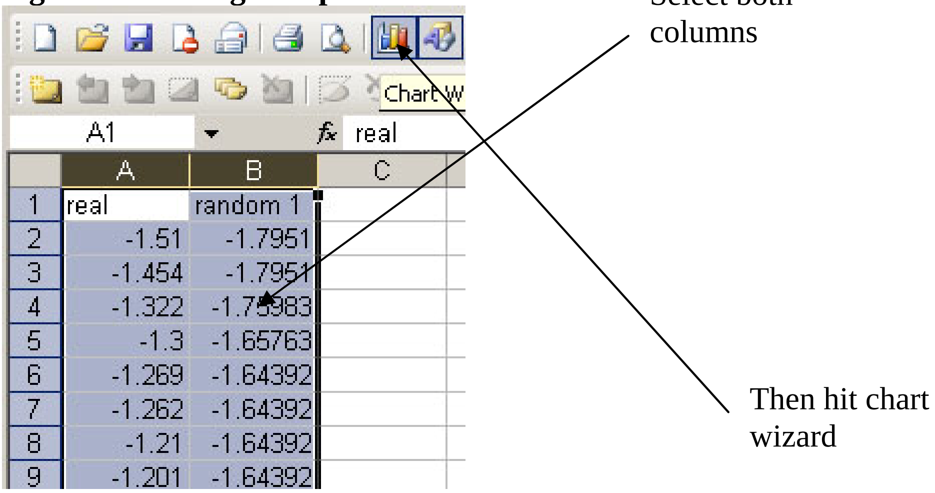

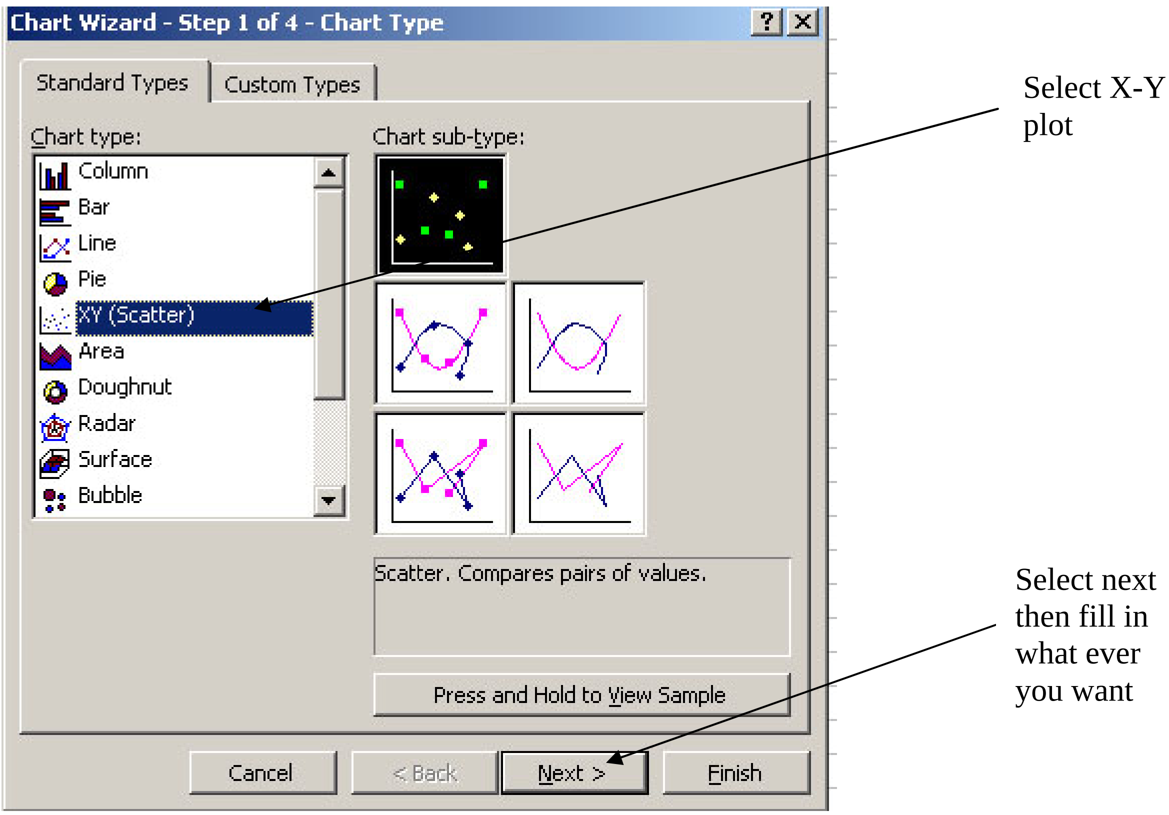

5. Interpret the plot! (Figure 10)
    a. Try drawing a line for the linear region of the plot? If there is not a linear region your data is not Gaussian. It may have a bimodal distribution depending on percentage of arrayed elements enriched in the IP. When you enrich greater then > 20 % of the arrayed elements the data distribution is more bimodal then normal.

b. Is there a heavy skew to the left? Are there many spots above the line in the bottom left of the chart? If there is a heavy skew on the left side of the distribution then the Gaussian assumption may be too liberal. Depending on how heavy the tail is you may want to use the permutation simulation approach.
c. Does the line intersect (0,0)? If not the data may need to be normalized or centered. A slight deviation < 0.05 from (0,0) is ok, but too much will invalidate the assumption of symmetry.

Figure 10. Q-Q plot in Excel

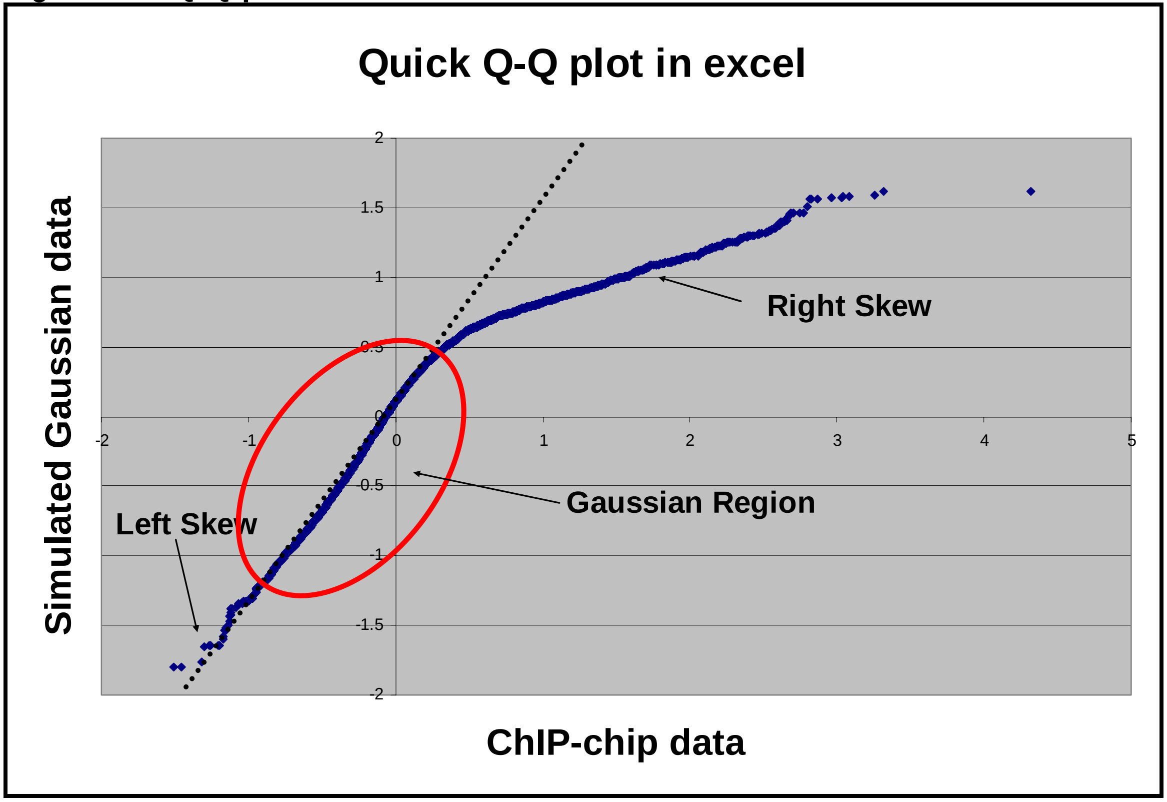

Supplement: Additional data file 2 — A PDF file containing detailed instructions for the installation and use of ChIPOTle. [file gb-2005-6-11-r97-S2.pdf]
